# A State-of-the-Art Review on the Evolving Utility of Endoscopic Ultrasound in Liver Diseases Diagnosis

**DOI:** 10.3390/diagnostics10080512

**Published:** 2020-07-23

**Authors:** Wisam Sbeit, Anas Kadah, Mahmud Mahamid, Rinaldo Pellicano, Amir Mari, Tawfik Khoury

**Affiliations:** 1 Department of Gastroenterology, Galilee Medical Center, Nahariya 22100, Israel; wisams@gmc.gov.il (W.S.); anas18kadah@msn.com (A.K.); 2 Faculty of Medicine in the Galilee, Bar-Ilan University, Safed 1311502, Israel; 3 Department of Gastroenterology, Sharee Zedek Medical Center, Jerusalem 9103102, Israel; mahmudmahamid@yahoo.com; 4 Gastroenterology Unit, Molinette Hospital, 10126 Turin, Italy; rinaldo_pellican@hotmail.com; 5 Gastroenterology and Endoscopy Units, The Nazareth Hospital, EMMS, Nazareth 16100, Israel; amir.mari@hotmail.com

**Keywords:** EUS, endoscopic ultrasonography, liver diseases, diagnosis

## Abstract

Liver diseases are amongst the most common diseases worldwide and manifest as a parenchymatic and/or biliary injury due to several causes as well as focal liver lesions, ranging from benign to malignant ones. The diagnosis of liver diseases is based mainly on biochemical and advanced imaging studies and, when required, on liver biopsy. Endoscopic ultrasound (EUS), which combines endoscopy and ultrasonography, is one of the main examination techniques used in gastroenterology as it is applied to evaluate abnormalities in the lumen of the upper and lower gastrointestinal tract and to define pancreatic and hepato-biliary features, often in chronic patients. Given its high spatial resolution and its proximity to the liver, EUS is gaining popularity in the diagnostic work up of liver diseases. This is a comprehensive overview of the current literature on the diagnostic indications for EUS use in patients with liver diseases. We performed a MEDLINE\PubMed and Embase search, and all articles that were relevant, after reviewing abstracts, were assessed and the full text was analyzed to extract data regarding technical success, diagnostic yield, bioptic characteristics, and complications rate. EUS-guided imaging and biopsy techniques in liver diseases have shown consistent favorable promising results among the reports through the literature, with an excellent diagnostic yield and safety profile, especially in the context of focal lesions and portal hypertension. The application of EUS in the diagnosis of liver diseases is a promising technique and should be considered as a first-line therapeutic option in selected cases.

## 1. Introduction

Since its first introduction in the 1980s [1], the applications of endoscopic ultrasound (EUS) are expanding quickly, due to its high spatial resolution to cover almost every field in gastroenterology, in addition to cardiac, renal, and the respiratory tract [2]. These diverse diagnostic and therapeutic applications have become feasible, taking advantage of the unique property of EUS, which combines endoscopy and ultrasonography (US). In recent years, an expanding volume of reports, describing the use of EUS as a complementary diagnostic tool in some contexts of liver diseases, has been published. In this review, we summarized the current data on EUS applications in hepatology.

## 2. Methods

A search for studies published before 30 March 2020 was performed using the Medline/PubMed and Embase databases inputting the keywords “EUS” or “endoscopic ultrasound” and any of the following: “liver” or “hepatic”, “portal hypertension”, “focal lesions”, “gastric” or “esophageal varices”, “cirrhosis”, “biopsy”, “fine needle aspiration”, “fine needle biopsy”, and “liver diseases”. Overall, we screened 48 papers; of them, 19 articles were excluded as they were review articles. The remaining 29 articles were included in the final analysis. The search was restricted to articles published in the English language.

## 3. Advantages of Endoscopic Ultrasound (EUS) in Evaluating Liver Diseases

There are several advantages of EUS compared to conventional images obtained by US and computed tomography (CT). The most important advantage is the proximity of the EUS transducer to the liver, and the ability to identify intervening structures and blood vessels [3]. Together with its low rate of adverse events, EUS constitutes a highly suitable modality for diagnosis and staging of primary malignant tumors and metastatic liver diseases [4]. The second advantage is the possibility to combine its images with those of the novel real-time elastography (RTE) that provide semi-quantitative measurements of liver parenchyma and focal lesion stiffness as documented by color images [5]. The third one is due to the fact that new-generation echoendoscopes are equipped with color, power, and pulsed Doppler, allowing identification of blood vessels, and evaluating portal hypertension, collateral vessels in portal hypertension, and intervening vessels during puncture [5,6]. The fourth one is the ability to obtain contrast-enhancement (CE) images, which improve the diagnostic performance in the case of focal lesions [7]. The last one is the ability to perform EUS-guided liver biopsy, which seems to be safer than the percutaneous route of biopsy, especially in patients with liver cirrhosis ant its accompanying coagulation disorder [8].

Hence, the additive value of EUS in the management of liver diseases is derived mainly from the enhanced imaging quality and the biopsy acquisition ability.

## 4. EUS-Guided Imaging Diagnosis of Liver Diseases

### 4.1. EUS-Guided Imaging in Chronic Liver Diseases/Cirrhosis

According to the European Association for the Study of the Liver (EASL) clinical practice guidelines, liver biopsy is the gold standard diagnostic test for cirrhosis, but as it is not without drawbacks, including its invasive nature, cost, complications, and sampling errors, several noninvasive tests based on tissue stiffness, such as US elastography, have emerged as a substitute avoiding these drawbacks [9]. This procedure has the advantage of demonstrating a liver parenchyma volume of about 100 times greater than that obtained by biopsy, and thus it is potentially less amenable to sampling error [10]. Noninvasive fibrosis markers, such as liver stiffness measurements (transient elastography (TE), Fibroscan, and RTE), have the ability to diagnose the liver fibrosis degree [11]. However, the performance of these tests by the transabdominal approach is suboptimal, especially in the case of obese patients and in those with ascites [9]. In these cases, EUS-guided liver stiffness assessment is advantageous and more efficacious, given the proximity of the transducer to the liver, thus avoiding the abdominal wall fat and interposed abdominal gas. Thus, EUS may serve as a more accurate test for liver fibrosis as measured by RTE [12]. By the proximity of the EUS transducer to the liver, EUS RTE is supposed to be more sensitive than transabdominal RTE in determining the liver fibrosis degree, due to the thinner gastric wall compared to abdominal the wall, which the signal has to penetrate [13]. The liver fibrosis index (LFI), calculated from RTE images, was shown to be correlated with advanced fibrosis according to the METAVIR score in chronic hepatitis C [14]. Based on the same principle, LFI obtained from EUS-RTE images was also shown to have a significant correlation with abdominal imaging and could discriminate between normal, fatty, and cirrhotic-appearing livers, and thus might be an adjunct tool in chronic liver disease investigation [13]. Transabdominal US elastography is the procedure of choice in assessing liver fibrosis, due to its ease of use, availability, low cost, and high safety profile. However, as most chronic liver disease patients will undergo upper endoscopy for variceal screening or follow up, and as in cases of liver enzymes abnormalities, investigation by EUS to rule out pancreato-biliary pathology will be performed, and the liver parenchyma may be evaluated by EUS elastography in the same session. A recent study by Tu et al. evaluated the accuracy of EUS, EUS-fibroscan, acoustic radiation force impulse, aspartate aminotransferase-to-platelet ratio, and their combination to detect varices. The authors included 322 patients with chronic viral liver disease and who were divided into an early cirrhosis group (Child–Pugh A grade) vs. a chronic hepatitis group based on clinical diagnosis. The authors reached the conclusion that these modalities had a significantly high detection rate in patients with early stage liver cirrhosis than in those with chronic hepatitis [15]. This finding underscores the emerging and promising role of EUS including fibroscan in reaching an early diagnosis of liver cirrhosis among patients with chronic hepatitis. Further studies are necessary to confirm the available data and to compare the other non-invasive fibrosis tools with EUS-based examination.

### 4.2. EUS-Guided Imaging in Focal Liver Lesions

**Figure 1:**
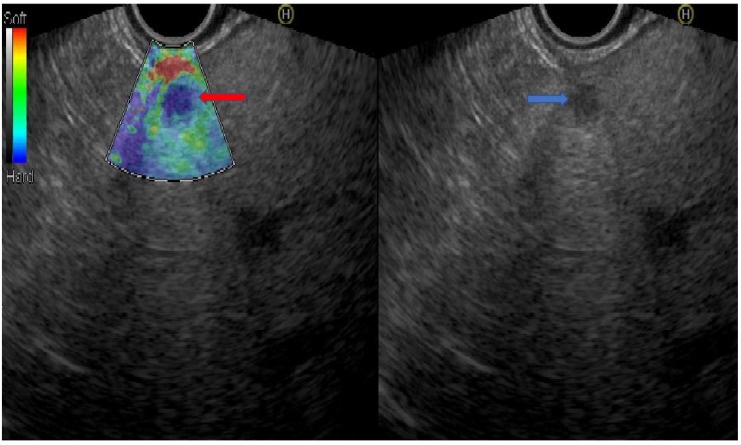
A patient with metastatic pancreatic adenocarcinoma to the liver. The blue arrow shows the liver metastasis by linear echoendoscope. The red arrow shows the lesion by real-time EUS-elastography in a blue color reflecting the hardness and stiffness of the lesion (the picture was supplied from the gastroenterology department at Galilee Medical Center).

Most focal liver lesions are incidentally diagnosed by US, CT, or magnetic resonance imaging (MRI) while sometimes these are discovered during surveillance of patients with high risk for hepatic malignancy or during a preoperative staging of malignancies. Elucidating the exact nature of these focal lesions is of tremendous significance as this may influence the management, stage, and prognosis [4]. Moreover, the traditional imaging studies may underestimate the diagnosis of small hepatic lesions. The diagnostic accuracy of EUS in detecting small hepatic lesions mostly less than 1 cm exceeds that of radiological examinations by US, CT, and MRI [4,16], and it may be used to detect suspicious small hepatic metastasis in the setting of other primary malignancies. To date, only few studies have assessed the additional benefit of traditional EUS over the other imaging tools for hepatic metastasis. An article by Awad et al. reported that EUS identified 28% of new additional hepatic lesions among 14 patients who were suspected to have hepatic metastasis by CT [17]. Moreover, another study addressed the diagnostic yield of EUS vs. CT in the detection of liver metastasis among patients with a newly confirmed diagnosis of pulmonary, pancreato-biliary, gastro-esophageal, and colonic malignancies. The diagnostic accuracies of EUS and CT scan for hepatic lesions were 98% and 92%, respectively (*p* = 0.05). Additionally, EUS detected a significantly higher number of metastatic lesions in the liver compared to CT (40 vs. 19, respectively; *p* = 0.008) [18]. Moreover, the advent of EUS elastography, which is the first non-invasive tool introduced to quantify the stiffness of liver tissue and liver masses [19], and its significant ability to distinguish between malignant and benign focal liver lesions, as the former are much more stiffer than the latter [20], has rendered available an additive tool to improve the EUS ability in the characterization of liver masses (Figure 1). For example, malignant liver masses are about 100-fold harder and stiffer than the surrounding normal tissue [20]. Furthermore, Saldolescu et al. demonstrated that benign lesions have significantly lower stiffness values than neoplastic lesions (hepatocellular carcinoma (HCC), cholangiocarcinoma, and metastases). The authors defined a hue histogram cut-off value of 170, which was able to discriminate between benign and malignant tumors with a 92.5% sensitivity, 88.8% specificity, 88.6% accuracy, 86.7% positive predictive value, and 92.3% negative predictive value [21]. Additionally, using ultrasound contrast agents (UCAs) either under Doppler by contrast-enhanced-EUS (CE-EUS) [22], or contrast harmonic-EUS (CH-EUS), the microvascular architecture could be visualized, allowing a better detection and characterization of focal lesions [22]. Taking advantage of the dual liver blood supply by the portal vein and hepatic artery, CH-EUS examination of the liver is divided into the arterial phase lasting up to 30 s from injection, during which enhancement increases progressively, the portal phase lasting from 30–120 s, and the venous phase thereafter [23]. The CE-EUS appearance of liver metastasis during the arterial phase shows variable contrast enhancements as they are mostly peripheral and weak hyper-enhance lesions. While, in the delay portal phase, the liver metastasis is demonstrated as dark hypo-vascular lesions. During both arterial and portal phases, hypo-enhancement is pathognomonic to all metastases, regardless of eventual enhancement in the arterial phase because the liver tissue retains the UCA, while the metastases present a rapid and marked “washout” [24].

A study by Oh et al. reported the additional usefulness of CH-EUS, over traditional EUS, to characterize suspected hepatic lesions in 30 patients. Before CE, 22 out of 30 patients (73.3%) were identified on traditional EUS. This number increased to 28 out of 30 patients (93.3%) after CE, with a 100% technical success and no procedure-related adverse events [25]. Thus, although data regarding the application of traditional EUS and CE-EUS are still accumulating, there is a promising benefit of introducing this diagnostic tool into the daily clinical practice in suspected cases to maximize the patient’s management. Further large prospective studies are warranted to confirm the aforementioned findings.

### 4.3. EUS-Guided Portal Pressure Measurement

The portal vein (PV) carries about 1500 mL/min of blood to the liver. Any increased resistance to flow may lead to portal hypertension. An increase in portal pressure induces the development of portosystemic collateral circulation with compensatory shunting, resulting in several disturbances, such as gastroesophageal varices. Cirrhosis, the end stage of any chronic liver disease, is the most important cause of portal hypertension. Since in patients with portal hypertension, prevention and therapy of the major complications, such as bleeding from gastroesophageal varices, are mandatory, the diagnosis of this condition represents a crucial step in hepatology. Portal pressure measurement can be accessed by two techniques. The first is by applying TE, which has also been shown to be an effective modality in evaluating portal hypertension. In asymptomatic patients with chronic liver disease, early portal hypertension could be detected by routine clinical data and TE [26]. A study, including patients with hepatitis C recurrence after liver transplantation showed that liver stiffness, as measured by TE, reflected with high sensitivity and specificity portal hypertension [27]. The second one is by performing direct measurement of portal pressure. In fact, EUS enjoys a high spatial resolution of blood vessels, thus allowing vascular interventions to be carried out, with the PV being the most attractive, especially in light of difficult standard percutaneous access [28]. This direct measurement aims to estimate the portal pressure gradient (PPG) by using a 25-gauge needle that is inserted into the PV and the hepatic vein (HV) or inferior vena cava (IVC), if HV is difficult to access, to estimate the direct pressure of both veins; then, PPG is derived by subtracting the HV pressure from the PV pressure. To date, only two human studies have been published on the utility of EUS-guided portal pressure measurement (Table 1). This technique was firstly reported by Fujii et al., who described a patient with recurrent gastrointestinal bleeding with PPG of 1 mm Hg without mention of procedure-related complications 4 days later [29]. An animal study, on swine, showed the effectiveness of this technique with excellent accuracy in estimating portal hypertension as it showed a strong correlation with the criterion standard of trans-jugular wedged and free HV pressure measurements by interventional radiology [30]. The second study, on humans, was performed by Huang et al., who reported 28 patients who underwent PPG measurement techniques with a complete technical success rate without adverse events; therefore, the procedure appears safe even in patients with portal hypertension [31]. Despite these encouraging data, prospective multicenter studies are needed to validate such results, before adopting this approach in the clinical setting.

### 4.4. EUS-Guided Varices Diagnosis

In cirrhotic patients, elevated liver stiffness as measured by TE could predict the presence of large esophageal varices [32]. The use of EUS in the diagnosis and management of gastric varices has expanded in the last decade. Gastric varices (GVs) are generally classified by the Sarin’s classification system [33], taking into account the location and direction of blood flow. The gastroesophageal varices 1 (GOV-1) is the most prevalent type and represents the extension of esophageal varices alongside the gastric lesser curvature. The GOV-2 type is the extension of esophageal varices along the greater curvature. The third and less common type is the isolated GV (IGV), consisting in isolated gastric varices localized in the upper gastric region, particularly the fundus. These GVs arise due to splenorenal or gastrorenal shunts. The GOV-1 type is generally associated with a lower risk of bleeding compared to GOV-2 and IGV, which represent the major source [34]. Several studies have established the advantage of EUS over upper gastrointestinal endoscopy in the recognition of all types of gastric varices [35]. Imamura et al. showed that the diameter of gastric varices was associated with flow volume, irrespective of the Child–Pugh class [36]. The addition of duplex and color Doppler EUS were assessed in a study including 20 volunteers and 11 patients with suspected splenic and/or portal thrombosis with possible shunts. The duplex and Doppler EUS provided a correct diagnosis in 10 out of 11 patients, whereas transabdominal US was unsuccessful to reach an accurate diagnosis in all patients. This finding shed light on the evolving role of EUS on the detection of splenic and portal veins thrombosis or a portosystemic shunt [37].

## 5. EUS-Guided Liver Biopsy

### 5.1. EUS-Guided Biopsy in Chronic Liver Diseases

Despite the great advance in the production of noninvasive tests intended to quantify liver fibrosis in patients with chronic liver diseases, their accuracy is inconclusive in a large proportion of cases, and thus liver biopsy is still the gold standard [9]. With our expanding experience and the continued progress in EUS-dedicated needles, tissue acquisition has improved to add another tool to our armamentarium [38]. Since its first description in 2007 [39], and compared with other modalities of liver biopsy, it was shown that EUS-guided liver biopsy (EUS–LB) using 19-gauge fine needle aspiration (FNA) needles is safe, with a yield comparable to or higher than percutaneous or transjugular biopsy [40]. To date, several studies have evaluated the diagnostic yield, accuracy, and safety profile of EUS-LB in patients with chronic liver diseases of various causes. In 2012, Stavropoulos et al. provided “proof of concept” that tissue acquisition with the regular 19-gauge EUS-FNA needle is adequate and that EUS-LB could be successfully performed with this needle [41]. Several years later, a prospective study comparing 19-gauge FNA versus 19-gauge fine needle biopsy (FNB) needles demonstrated a better performance of the core biopsy needle concerning the biopsy length and number of complete portal triads [42]. On the other hand, a prospective study comparing 19-gauge FNA with 22-gauge FNB showed higher tissue adequacy of the 19-gauge FNA in terms of sample length and less sample fragmentation [43]. However, in a recent meta-analysis of studies using 19-gauge needles, tissue acquisition was better in the FNA needles compared with the core biopsy needles, with a diagnostic yield of 95.8% and adverse events rate of 0.9% [44]. The superiority of the 19-gauge FNA needle in terms of specimen adequacy compared with the 22-gauge FNB needle and two types of 19-gauge FNB needle (one true cut and the other non-true cut) was also shown in a very recent study by Patel et al. [45]. A meta-analysis by Khan et al. summarized that cytological assessment of material obtained by FNA is proven to be sufficient; however, in cases needing tissue architecture examination, immunohistochemical staining, and molecular analysis, FNB is preferred to allow tissue acquisition for histologic examination [46]. The most prominent advantages of EUS-LB are that it relies on minor pain intensity as it does not include skin puncture, with image guidance thus ensuring blood vessel avoidance, providing access to all liver parenchyma including the entire left lobe and the majority of the right lobe. This allows quick and safe multiple liver passes from both lobes, thus decreasing histologic variability and providing same-session inspection of neighboring structures and lymph nodes and screening for varices [47]. As most US-guided, CT-guided, and transjugular liver biopsies are obtained from the right lobe, and as several studies reported variations in disease activity and staging in different liver lobes, a study by Khurana et al. showed that EUS-guided bi-lobar liver biopsy improved disease activity and fibrosis assessments. They concluded that EUS guidance enables sampling of both liver lobes at the same session and can be readily applied in clinical practice [48]. A recent review by Mok et al. has shown that EUS-LB is a safe and effective modality for liver core acquisition [49]. A systematic review by Wang et al. reported an EUS-FNA-related morbidity and mortality of 0.98% and 0.02%, respectively [50]. Table 2 and Table 3 show all studies addressing the utility of EUS-guided liver biopsy in parenchymal liver diseases. Overall, we could identify 16 papers including 913 patients. Among them, seven were retrospective studies and cases series, two were case reports, and the others were prospective non-randomized, prospective case series, and randomized controlled trials. The average technical success and diagnostic yield for EUS-FNA and EUS-FNB-guided liver biopsy were 100% and 89.8%, respectively, while complications occurred in 30 patients, yielding a complications rate of 3.3%, all of which were minor. Further comparison between the EUS-FNA and EUS-FNB groups revealed no difference in terms of technical success (100% for both), diagnostic yield of 97.4% for EUS-FNA vs. 87.6% for EUS-FNB (*p* = 0.09), average median specimen length of 31.5 mm for EUS-FNA vs. 30.8 for EUS-FNB (*p* = 0.4), average median complete portal tracts of 11.2 for EUS-FNA vs. 17.1 for EUS-FNB (*p* = 0.2), and similar average median needle passes of 2.2 for EUS-FNA vs. 2 for EUS-FNB (*p* = 0.3). However, there was a trend towards more procedure-related complications in the EUS-FNB studies compared to EUS-FNA studies (29/592 patients, 4.9%, vs. 1/321 patients, 0.3%, *p* = 0.07). Notably, data regarding the safety profile of EUS-LB in cirrhotic patients are lacking, thus more prospective studies with large sample sizes are needed. Moreover, there are no available studies that have assessed the cut-off levels of the coagulation profile and platelets counts permitting the safe performance of EUS-LB. Hence, more studies are needed to address these issues also before setting guideline recommendations.

### 5.2. EUS and Non-Alcoholic Fatty Liver Disease

In Western countries, non-alcoholic fatty liver disease (NAFLD), with its evolutive form called nonalcoholic steatohepatitis (NASH), represents one of the major causes of chronic liver disease [51]. NAFLD is usually suspected by the combination of abnormal liver transaminases, hyperechoic liver on US, and the absence of heavy alcohol drinking and any other chronic liver disease cause [48]. The staging of fibrosis by the noninvasive fibroscan is suboptimal, especially in stages F2 and F3 [52]. Hence, LB remains a gold standard in diagnosing the advanced forms (F3) [53]. As the number of NASH patients and the expanding potential pharmacologic therapies for this condition are increasing, it seems that the need for LB for disease staging will also increase [54]. Because EUS-LB enjoys US guidance, this may improve its yield by directing the needle to the fattiest area [55]. To date, only two studies have reported the utility of EUS-LB in a cohort of patients with NAFLD (Table 4). The largest cohort was evaluated by Saab et al. and includes 47 patients with fatty liver who underwent EUS-FNB with 19-gauge SharkCore needle biopsy with high diagnostic yield and technical success, while only two patients developed a minor self-limited liver hematoma that resolved with conservative management [56]. Another study, performed by Bazerbachi et al., reported a similar efficacy and safety rate among 21 patients with NAFLD using a 22G SharkCore FNB needle, with minimal adverse events occurring in six patients, which were all limited to mild post-procedural pain [55]. We analyzed all studies that were performed in patients with chronic liver diseases (Table 2 and Table 3), extracting the data regarding the NAFLD subgroup patients alone from the entire study cohort and analyzed their data independently. We identified five studies, as shown in Table 4. All studies reported an excellent technical success, diagnostic yield, and zero complications rate [57], suggesting the high efficacy and safety of both needle types, FNA and FNB, in obtaining LB via EUS. Notably, all other studies reported in Table 2 and those that assessed the EUS-LB in chronic liver diseases were not included into our analysis shown in Table 4 given that they lack independent analysis of the NAFLD patients’ subgroup. Overall, we could identify seven papers (overall, 103 patients), all of which were case series studies, where four of them had a retrospective design while the rest had a prospective design. The average technical success and diagnostic yield were 100% and 96.8%, respectively, while complications occurred in 8 patients, yielding a complications rate of 7.7%, all of which were mild.

### 5.3. EUS and Focal Liver Lesions

As tissue diagnosis is usually needed in most liver lesions and especially in otherwise indeterminate hepatic solid masses, needle biopsy is the next step. Not infrequently, immunohistochemical staining may be needed to allow differentiation between different hepatic solid masses where cytological evaluation alone may not be sufficient. To date, several studies have reported the diagnostic yield of EUS-FNA/FNB in liver masses. The largest study was performed by TenBerge et al. on 167 cases in which EUS-FNA of the liver was performed, and showed an excellent overall diagnostic yield of 95.8% for malignant and benign liver lesions, as the findings of the cytopathology was malignancy in 138 (82.6%), benign in 22 (13.2%), and indeterminate in 7 (4%), with a 3.6% complication rate [66]. Moreover, a retrospective analysis evaluating the accuracy of EUS-FNB in hepatic solid masses showed a high diagnostic accuracy (89.7%), sensitivity (89.7%), specificity (100%), and sample adequacy (91.4%) for histology [67]. A recent review by Ichim et al. reached the conclusion that EUS-FNA of focal hepatic lesions is comparable, if not superior, to US/CT-guided biopsy, with a diagnostic yield of 80–100% [68]. Although this data is still emerging, according to the available literature, both needle types (FNA and FNB) have a similar efficacy and safety profile. Overall, we could identify nine papers, including six prospective and three retrospective studies (overall 463 patients) (Table 5). The average diagnostic yield was excellent, approaching 94.8%, while only 7 patients had complications, yielding a complications rate of 1.5%. Still, more prospective comparative studies are needed to more precisely assess the performance of FNA and FNB needles.

## 6. Conclusions

This interesting rapidly evolving field of EUS in liver diseases diagnosis has induced a big step in several aspects, including improved visualization of liver lesions and better tissue acquisition of diffuse and focal hepatic lesions, in addition to measuring portal pressure and diagnosing gastric varices, due to its high spatial resolution, proximity of the transducer to the organ of interest, and minimal invasiveness and excellent safety profile. This state-of-the-art review demonstrates the promising evolving diagnostic potential of this technique, as it was shown to be consistently effective with a high safety profile across the various studies reported. Probably, in the next years, some of our first-line recommended approaches will change in favor of these rapidly growing and expanding applications to cover almost all aspects of diagnostic hepatology.

## Figures and Tables

**Table 1:** Human studies reporting the utility of EUS-guided portal hypertension measurement.

Reference	Patients No.	Technical Success, %	Diagnostic Yield, %	Needle Used	PPG mm Hg (Mean, Range)	Complications, N (%)
Fujii et al. [29] _Case report_	1	100	100	FNA/22G	1	None
Huang et al. [31] _Prospective_	28	100	100	FNA/25G	8.2 (1.5–19)	None

**Table 2:** Studies reporting the efficacy and safety of EUS-FNA-guided liver biopsy in patients with chronic liver disease.

Reference	Patients No.	Technical Success (%)	Diagnostic Yield (%)	Specimen Length in mm (Median, Range)	Complete Portal Tracts (Median, Range)	Complications, N (%)	Needle Passes (Median)	Needle Used/Size
Pineda et al. [40] _Retrospective study_	110	100	98	38 (24–81)	14 (9–27)	0	-	FNA/19G
Shuja et al. [47] _Retrospective study_	69	100	100	45.8 (mean)	10.84 (mean)	0	3	FNA/19G
Stavropoulos et al. [41] _Prospective case series_	22	100	91	36.9 (2–184.6)	9 (1–73)	0	2 (1–3)	FNA/19G
Diehl et al. [58] *_Prospective non-randomized_*_study_	110	100	98	38 (0–203)	14 (0–68)	1 (0.9) ^a^	1.5 (1–2)	FNA/19
Gor et al. [59] _Retrospective case series_	10	100	100	13 (6–23)	8 (6–15)	0	-	FNA/19G

^a^ Mild bleeding with thrombocytopenia and coagulopathy (1 patient).

**Table 3:** Studies reporting the efficacy and safety of EUS-FNB-guided liver biopsy in patients with chronic liver disease.

Reference	Patients No.	Technical Success (%)	Diagnostic Yield (%)	Specimen Length in mm (Median, Range)	Complete Portal tracts (Median, Range)	Complications, N (%)	Needle Passes (Median)	Needle Used/Size
Shah et al. [57] _Retrospective study_	24	100	96	65.6 (17–167.4)	32.5 (5–85)	2 (8.3) ^b^	2 (1–3)	SharkCore/19G
Nieto et al. [60] _Retrospective study_	165	100	100	60 (43–80)	18 (13–24)	3 (1.8) ^c^	1	SharkCore/19G
Mathew et al. [39] _Case report_	2	100	100	-	-	0	-	Quickcore/19G
Ching et al. [42] _Prospective randomized trial_	20 20	100 100	100 100	114 (mean) 153.2 (mean)	16.5 (6–38) 38 (0–81)	8 (40) ^d^ 7 (35) ^d^	- -	FNA/19 Acquire/19G
Mok et al. [43] _Prospective randomized trial_	40 40	100 100	88 68	- -	- -	0 1 (2.5) ^e^	- -	FNA/19G Sharkcore/22G
Patel et al. [45] _Retrospective study_	30 50 28 27	100 100 100 100	66.7 46 82.1 81.5	I.8 (mean) 4.7 (mean) I.9 (mean) 8.4 (mean)	6.9 (mean) 3 (mean) 7.3 (mean) 16.9 (mean)	- - - -	- - - -	Acquire/22G Quickcore/19G Procore/19G Expect/19G
Gleeson et al. [61] _Retrospective study_	9	100	100	13 (8–28)	7 (5–8)	0	2 (1–3)	Quickcore/19
DeWitt et al. [62] _Prospective case series_	21	100	90.5	9 (1–23)	2 (0–10)	0	3 (1–4)	Quickcore/19
Nakai et al. [63] _Case report_	1	100	100	15	8	0	1	ProCore/19
Sey et al. [64] _Prospective cross-sectional study_	45 30	100 100	73.3 96.7	9 (0–25) 20 (5–60)	2 (0–15) 5 (0–24)	2 (4.4) ^f^ 0	3 2	Quickcore/19 ProCore/19
Hasan et al. [65] *_Prospective non-randomized_*_study_	40	100	100	55 (44.5–68)	42 (28.5–53)	6 (15) ^g^	-	Acquire/22G

^b^ Mild abdominal pain (1) and subcapsular bleeding (1); ^c^ Abdominal pain (2) and self-limited hematoma (1); ^d^ Mild abdominal pain (15); ^e^ abdominal pain (1); ^f^ Mild abdominal pain (2); ^g^ Self-limited abdominal pain (6).

**Table 4:** Studies reporting the efficacy and safety of EUS-guided liver biopsy in non-alcoholic fatty liver disease.

Reference	Patients No.	Technical Success (%)	Diagnostic Yield (%)	Specimen Length in mm (Median, Range)	Complete Portal Tracts (Median, Range)	Complications, N (%)	Needle Passes (Median)	Needle Used/Size
Saab et al. [56] _Retrospective case series_	47	100	100	65 (46-80)	18 (14–24)	2 (4.2) ^a^	1	SharkCore/19G
Bazerbachi et al. [55] _Prospective blinded trial_	21	100	100	24 (20–27.5)	26 (7–62)	6 (7) ^b^	2	SharkCore/22
Dewitt et al. [62] _Prospective case series_	9	100	77.8	8 (1–13)	2 (0–9)	0	3	Quickcore/19G
Gleeson et al. [61] _Retrospective case series_	6	100	100	11.5 (8–27)	7 (5–8)	0	2 (1–3)	Quickcore/19G
Gor et al. [59] _Retrospective case series_	4	100	100	11 (6–23)	6.5 (6–14)	0	-	FNA/19G
Stavropoulos et al. [41] _Prospective case series_	5	100	100	32.2 (12.5–58.7)	9 (4–13)	0	1	FNA/19G
Shah et al. [57] _Retrospective_	11	100	100	71.1 (17.1–167.4)	33 (23–85)	-	2 (1–3)	SharkCore/19G

^a^ Self-limited liver hematoma that resolved with conservative management (2 patients); ^b^ Postprocedural pain (6).

**Table 5:** Studies reporting the utility of EUS-guided liver biopsy in focal liver lesions.

Reference	Patients No.	Diagnostic Yield (%)	Complications, N (%)	Needle Passes, Median	Needle Used/Size
Ichim et al. [4] _Prospective study_	48	98	0	2	FNA/22G
Sing et al. [18] _Prospective study_	26	98	0	2.1	FNA/22
TenBerge et al. [66] _Retrospective study_	167	95.8	6 (3.6) ^a^	-	FNA/-
Chon et al. [67] _Retrospective study_	58	89.7	1 (1.7) ^b^	2	FNB ProCore/20 or 22 or 25G
Nguyen et al. [69] _Prospective study_	14	100	0	2	FNA/22G
DeWitt et al. [70] _Retrospective study_	77	91	0	3.4 (mean)	FNA/22
McGrath et al. [71] _Retrospective study_	5	100	0	2	FNA/22
Lee et al. [72] _Prospective study_	21	90.5	0	2	FNB/20G or 22G or 25G
Oh D. et al. [73] _Prospective observational study_	47	90.5	0	3	FNA/22 or 25

^a^ Death in 1 patient with an occluding biliary stent and biliary sepsis, mild bleeding (1), fever (2), and pain (2); ^b^ Bleeding complication, which was controlled with endoscopic hemostasis.

## Abbreviations

EUS	endoscopic ultrasound
US	ultrasound
CT	computed tomography
RTE	real-time elastography
CE	Contrast-Enhancement
EASL	European association for the study of liver disease
TE	transient elastography
LFI	Liver fibrosis index
MRI	magnetic resonance imaging
UCA	ultrasound contrast agents
CE-EUS	contrast-enhanced-EUS
CH-EUS	contrast harmonic-EUS
PPG	portal pressure gradient
PV	portal vein
HV	hepatic vein
IVC	inferior vena cava
GV	Gastric varices
GOV	gastroesophageal varices
IGV	isolated gastric varices
EUS-LB	endoscopic ultrasound-guided liver biopsy
FNA	fine needle aspiration
FNB	fine needle biopsy
NAFLD	non-alcoholic fatty liver disease
NASH	nonalcoholic steatohepatitis
